# On the Stator Slot Geometry of a Cable Wound Generator for Hydrokinetic Energy Conversion

**DOI:** 10.1155/2015/812149

**Published:** 2015-03-23

**Authors:** Mårten Grabbe, Sandra Eriksson, Mats Leijon

**Affiliations:** The Swedish Centre for Renewable Electric Energy Conversion, Division of Electricity, The Ångström Laboratory, Uppsala University, P.O. Box 534, 751 21 Uppsala, Sweden

## Abstract

The stator slot geometry of a cable wound permanent magnet synchronous generator for hydrokinetic energy conversion is evaluated. Practical experience from winding two cable wound generators is used to propose optimized dimensions of different parts in the stator slot geometry. A thorough investigation is performed through simulations of how small geometrical changes alter the generator performance. The finite element method (FEM) is used to model the generator and the simulations show that small changes in the geometry can have large effect on the performance of the generator. Furthermore, it is concluded that the load angle is especially sensitive to small geometrical changes. A new generator design is proposed which shows improved efficiency, reduced weight, and a possibility to decrease the expensive permanent magnet material by almost one-fifth.

## 1. Introduction

The permanent magnet geometry and stator slot geometry are important design parameters in the magnetic circuit of an electrical machine. In addition, utilizing cable winding presents new stator slot geometries that have not previously been studied in great detail. In this paper, the stator slot design of a cable wound permanent magnet synchronous generator for hydrokinetic energy conversion is studied using finite element (FE) simulations. The importance of changes on the millimetre scale in the stator slot geometry is discussed based on the practical experience from the design and the assembly of two prototypes.

The generator is designed to be directly connected to a fixed pitch vertical axis turbine, operating at both variable speed and power to electrically control the tip speed ratio of the turbine [1]. The generator is also intended to efficiently brake the turbine at water velocities above nominal operation, implying that a low load angle at nominal operation is preferable. Other direct drive designs for tidal turbines have been proposed recently; see, for instance, [2–6], but as the focus is on the stator slot geometry, the work presented here is more closely related to studies on other cable wound machine designs for wind power [7], wave power [8], and the high voltage Powerformer [9].

The starting point, or reference geometry, is that of the first prototype presented in [10], rated at 5 kW and 150 V at 10 rpm. As with most early prototypes, the generator design at hand can most likely be improved in many aspects. In this study, however, the focus is on possible performance gains by changing the stator slot geometry in the existing design. In order to incorporate practical experience from construction of two prototypes, the influence of one parameter at a time is discussed rather than a traditional optimization study. Such improvements would likely be relevant even if other parameters in the design would be changed.

## 2. Reference Machine Characteristics

**Figure 1:**
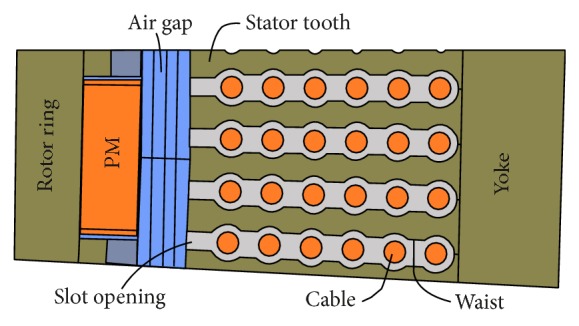
The geometry used in the FE model of the reference machine.

The 5 kW reference machine has 120 poles and is designed for the low velocities presented by tidal currents. Surface mounted Nd_2_Fe_14_B magnets are used and the stator is stacked with laser cut M800-100A sheets with a single row of six 16 mm^2^ cables in each slot as seen in Figure 1.

The magnet width and slot opening design of the reference machine have largely evolved from a previous study of a cable wound linear generator [8]. In this case, however, the focus is on the interior geometry of the stator slots.

At first, it should be noted that the cable windings are not inserted radially from the air gap. Rather, the cable is inserted axially into each stator slot position and wound in a fractional (7/5) wave winding. No slot wedges are used, but instead the stator slot opening is narrow to prevent the cables from entering the air gap. Furthermore, a small waist is introduced between each cable to prevent them from rubbing against each other during the winding procedure and to keep them fixed in place during operation.

**Figure 2:**
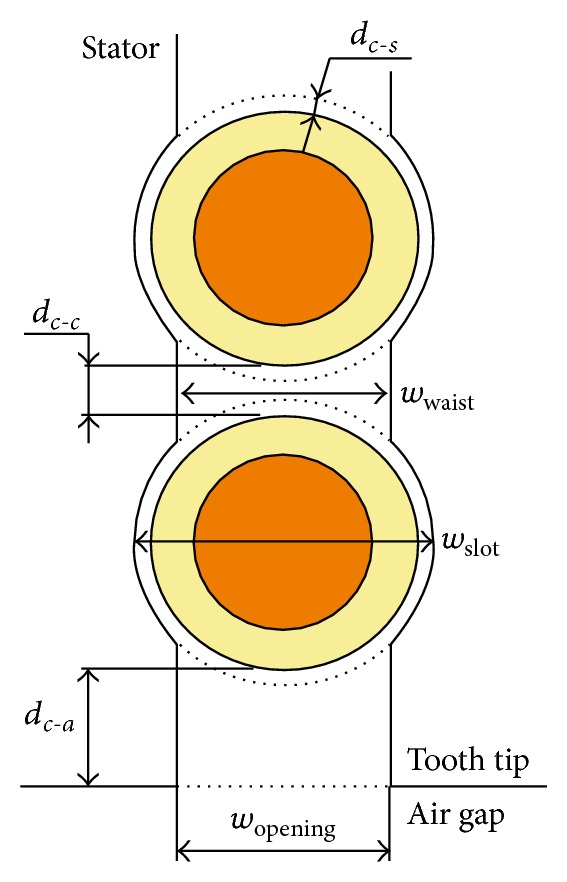
A stator slot opening and the two cables closest to the air gap showing the geometry parameters used in this study.

The main characteristics of the reference generator are presented in Table 1. The stator slot geometry is detailed in column one of Table 2 and Figure 2, and the resulting performance is summarized in the first column of Table 3.

## 3. FE Model of the Generator

The generator is designed with the aid of an in-house developed design tool and the combined set of field and circuit equations are solved in the finite element environment ACE [11]. The magnetic field inside the core of the generator is assumed to be axisymmetrical and modeled in two dimensions. The displacement field is neglected and the permanent magnets are modeled using the current sheet approach [12]. Furthermore, coil end impedances are introduced in the circuit equations, the laminated stator core is modeled using a singl-valued magnetization curve, and a correction factor of 1.5 is used for all iron losses.

The model used is described in more detail in [13] and simulations of the first prototype have been compared to experiments in [10]. The mesh includes around 33000 elements and second-order shape functions are used.

## 4. Simulations of the Stator Slot Geometry

The parameters that are changed are the the waist (*w*_waist_), the distance between cables (*d*_*c*−*c*_), the distance between cable and air gap (*d*_*c*−*a*_), and the distance between cable and stator (*d*_*c*−*s*_), as shown in Figure 2.

Simulations are performed using the model presented in Section 3. One parameter at a time is changed, while all other parameters are fixed to their reference value. All computations are performed at nominal load and nominal speed. If the slot depth is changed, the stator outer diameter is adjusted accordingly to maintain the same yoke width. As the voltage and power are kept fixed, changes in the axial length are allowed. The changes are a few millimetres at most and are only presented indirectly through small changes in copper losses, iron losses, and stator weight.

The generator is intended to be used with a diode rectifier. In the simulations, the generator is connected to a purely resistive load, which corresponds to a power factor of unity as is the case with diode rectification. During the analysis, the voltage and power are kept constant. According to the circuit theory, the load angle, *δ*, for a generator with unity power factor can be calculated as sin(*δ*) = *XI*/*E*, where *X* is the synchronous reactance, *I* is the current, and *E* is the internal voltage. The change in load angle can therefore be seen as a direct measurement of change in the machine reactance.

### 4.1. Waist Design

In the reference case, the slot width is 8 mm and the waist is 6 mm at its narrowest point. The waist factor, defined as *w*_waist_/*w*_slot_, is thus 0.75 in the reference geometry.

**Figure 3:**
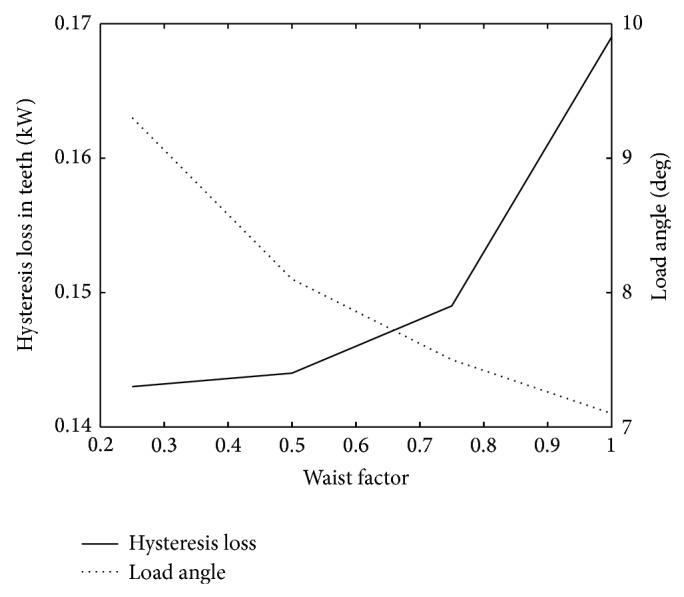
The hysteresis loss in the stator teeth and the load angle as a function of the waist factor.

A pronounced waist keeps the cable firmly in place and reduces the area in the tooth with high magnetic induction, thereby slightly reducing the iron losses. However, the waist also increases the leakage flux resulting in a higher load angle. As can be seen in Figure 3, no waist decreases the load angle but increases the hysteresis losses in the stator tooth. Without a waist, however, some other means of protecting the cables from rubbing against each other during winding would have to be implemented.

### 4.2. Cable to Cable

The distance between cables (*d*_*c*−*c*_) may have practical considerations during the winding procedure. Keeping the cables close to each other results in shorter stator teeth and a more compact design. However, if the cables are positioned* too* closely, a more pronounced waist may be required to prevent them from rubbing against each other during winding. The coil end positioning may also be affected by the distance between cables. A distance of 2 mm was used successfully in the reference machine, making a more compact design look viable.

**Figure 4:**
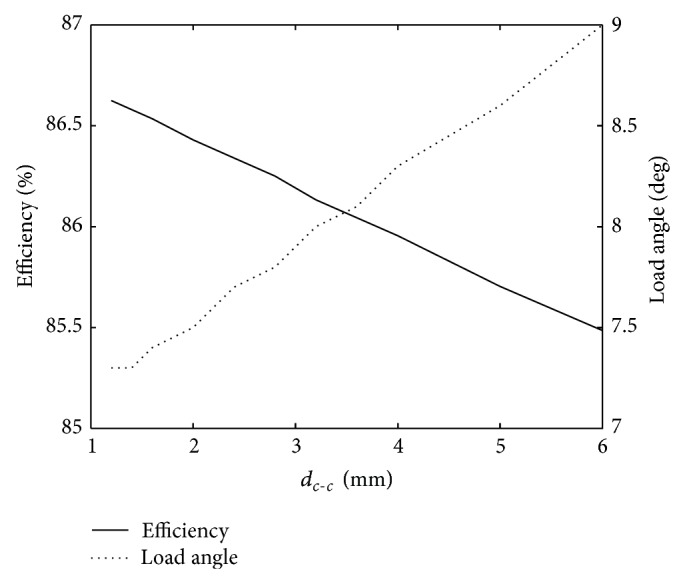
The efficiency and the load angle as a function of the distance between cables.

Gains in both efficiency and load angle can be achieved by moving the cables closer to each other (see Figure 4). Perhaps more importantly, the stator weight and stator outer diameter can be reduced by 4% and 8 mm, respectively. It should be noted though, that the same space would still be required for the coil ends.

### 4.3. Cable to Air Gap

Moving the winding closer to the air gap is perhaps the most straight forward way to achieve a more compact design. One might have to consider, depending on winding scheme, if the coil ends need a certain space to the air gap. A distance of 5.5 mm from the air gap to the first cable position was chosen in the reference machine. It turned out to work well in practice, as the coil ends could be pushed backwards away from the air gap, leaving room for improvements in this area as well.

**Figure 5:**
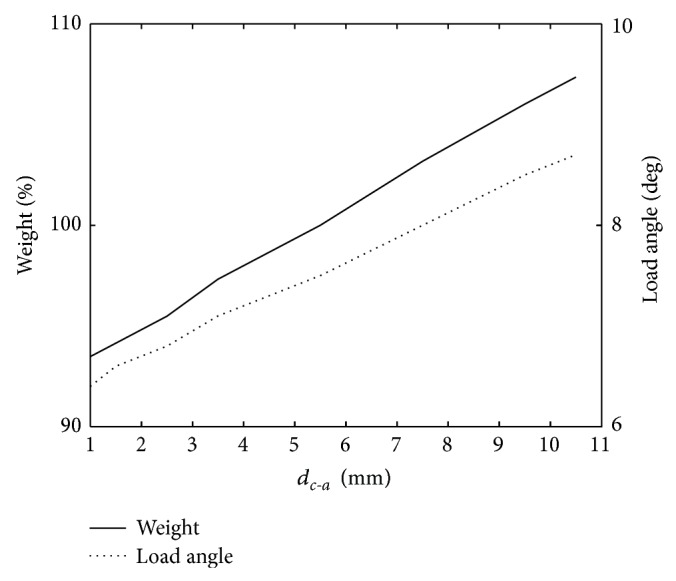
The change in weight and the load angle as a function of the distance from the winding to the air gap.

The distance to the air gap hardly affects the efficiency at nominal load conditions. However, as can be seen in Figure 5, the weight of the machine and load angle are both decreased as the windings are moved closer to the air gap.

### 4.4. Slot Width

The distance between the cable and the stator (*d*_*c*−*s*_) might be the single parameter that most clearly affects the winding procedure. Assuming the stator sheets are well aligned and properly stacked, a certain distance between the cable and the stator is still needed to allow for a smooth and easy winding procedure. A too tight design might cause unnecessary wear on the cables.

Cables with a diameter of 7 mm were used to wind the reference machine with a slot width of 8 mm (i.e., *d*_*c*−*s*_ = 0.5 mm). The winding procedure went smoothly. It should however be noted that the prototype was wound by hand, and the distance between cable and stator might have to be reevaluated when utilizing industrial robots for automated winding [14].

**Figure 6:**
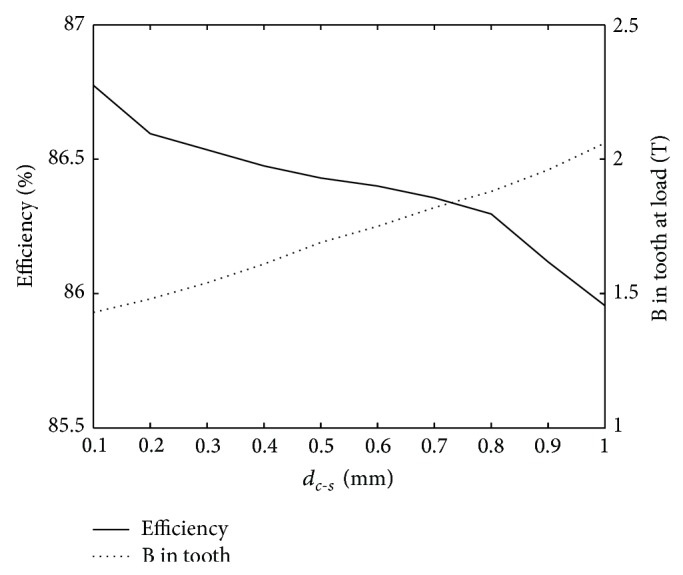
Efficiency and B-field in tooth at nominal load for varying distance between cable and stator.

The slot width is changed, which results in a new tooth width. Thus, clear changes are seen in the B-field in the stator tooth as *d*_*c*−*s*_ is changed (see Figure 6). In other words, if a larger gap between cable and slot is needed, the design will also have to incorporate a slightly larger stator diameter to accommodate sufficiently wide stator teeth.

## 5. Results and Discussion

Incorporating all the small changes in the geometry discussed above may add up to a significant improvement. However, none of the predicted improvements would be worthwhile if it turned out to complicate the manufacturing and the assembly procedure.

Winding the reference machine went smoothly, most likely thanks to proper stacking and good alignment of the stator sheets. The result from the reference machine has validated the results of the simulations as presented in [10]. This experience gave confidence to incorporate some changes in the second prototype [15]. There, the winding was moved closer to the air gap (2.4 mm instead of 5.5 mm), the cables were moved closer to each other (1 mm instead of 2 mm), and the gap between cable and stator was decreased to 0.4 mm. Again, the winding procedure went smoothly without appreciable wear on the cable insulation.

**Figure 7:**
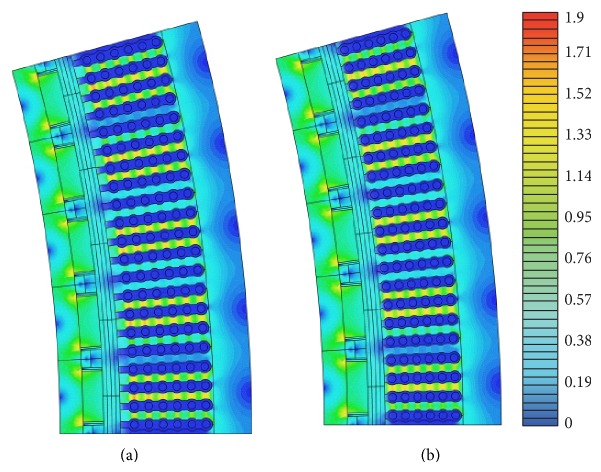
Illustration of the magnetic field distribution in the reference machine (to the left) and the new design (to the right).

Experience from winding the second prototype has given confidence that further incremental changes to the geometry may be implemented in practice. A tighter gap between cable and stator (0.3 mm) would render a less pronounced waist possible. Experience also indicate that the cables could be moved closer to the air gap (1.3 mm), further decreasing the slot depth and the generator outer diameter. The new stator slot design is shown in Figure 7 and compared to the reference machine in Tables 2 and 3. The proposed changes would, according to simulations, improve the efficiency from 86.4% to 87.0% and lower the load angle from 7.5° to 6.2°. Perhaps more significantly, the stator weight would be decreased by 11%, and the outer diameter would be decreased by 20.4 mm allowing for further savings on the support structure.

The decrease in load angle can be translated into either a decrease in magnetic material necessary to maintain the nominal design point of 150 V and 5 kW at a load angle of 7.5°, or an increase in power output at the same voltage and load angle as the reference machine. Simulations predict that the new design can reach the nominal design point utilizing 19% less permanent magnet material, or a 22% increase in electrical power output at the reference voltage and load angle.

## 6. Conclusions

The finite element analysis of the cable wound generator shows that the performance can be improved by apparently small changes in the stator slot geometry. A new design is proposed based on practical experience from winding two prototypes, and simulations predict an increase in efficiency of 0.6 percentage points, while the stator core weight is reduced by 11% and the load angle is decreased by 17%. The more compact design allows for further savings on the support structure. The decrease in load angle can be translated into a 19% decrease in magnetic material at the nominal design point. Finally, it can be concluded that the load angle is influenced by small alterations in the stator geometry. Therefore, a thorough evaluation of the stator slot geometry is important when optimizing a generator design.

## Figures and Tables

**Table 1:** Reference machine main design parameters.

Power	5 kW
Voltage	150 V
Rotational speed	10 rpm
Number of poles	120
Fractional winding	7/5
Stator outer diameter	2000 mm
Stator inner diameter	1835 mm
Air gap	10.5 mm
Machine length	270 mm
Magnet height	13 mm
Magnet width	32 mm
PM remanence	1.22 T

**Table 2:** Stator slot geometry of the reference machine compared to the suggested improved geometry.

Parameter	Reference	New design
Slot opening	4 mm	4 mm
Slot width (*w*_slot_)	8 mm	7.6 mm
Cable diameter	7 mm	7 mm
Waist width (*w*_waist_)	6 mm	6.1 mm
Waist factor	0.75	0.8
Cable to cable (*d*_*c*–*c*_)	2 mm	0.8 mm
Cable to air gap (*d*_*c*–*a*_)	5.5 mm	1.3 mm
Cable to stator (*d*_*c*–*s*_)	0.5 mm	0.3 mm
Slot depth	58 mm	47.6 mm

**Table 3:** Reference machine performance at its nominal operating point compared with the suggested improved design.

Parameter	Reference	New design
Efficiency	86.4%	87.0%
B in stator tooth	1.69 T	1.57 T
Hysteresis losses in teeth	0.149 kW	0.128 kW
Eddy current losses in teeth	0.037 kW	0.032 kW
Total losses	0.785 kW	0.748 kW
Load angle	7.5°	6.2°
Stator weight	598 kg	532 kg
